# Immediate effects of rest periods on balance control in patients after stroke. A randomized controlled pilot trial

**DOI:** 10.1186/s13104-018-3450-2

**Published:** 2018-05-24

**Authors:** Bernhard Elsner, Simon Schweder, Jan Mehrholz

**Affiliations:** 1 Department of Public Health, Medical School, Technical University Dresden, Dresden, Germany; 2 SRH Hochschule Gera, Gera, Germany; 3 Wissenschaftliches Institut, Private Europäische Medizinische Akademie der Klinik Bavaria in Kreischa GmbH, An der Wolfsschlucht 1-2, 01731 Kreischa, Germany

**Keywords:** Stroke, Balance, Rest, Rehabilitation, Physiotherapy

## Abstract

**Objectives:**

This randomized controlled trial evaluates the effects of two different rest periods between as set of balance exercises after stroke during inpatient rehabilitation.

**Results:**

Twenty patients after stroke [11 males; mean (SD) age 65.4 (11.5) years; duration of illness 5.3 (3.4) weeks; 16 (80%) left-sided strokes] were randomly allocated into two groups of either a full rest (FR) of 4 min (n = 10) or a short rest (SR) of 1 min between exercise sets (n = 10). Patients improved from baseline until immediately after exercises in one-leg standing time on the affected leg [SR: mean difference 5.1 s (SD 10.3) and FR: 2.0 s (2.4)] and tandem standing time (TST). [SR: 14.9 s (SD 24.6) and FR: 5.7 s (12.0)], but OLST and TST did not differ significantly between groups (p = 0.35 and p = 0.52, respectively).

*Trial registration* The study was registered retrospectively in the German Register of Clinical Trials with the ID: DRKS00013979

**Electronic supplementary material:**

The online version of this article (10.1186/s13104-018-3450-2) contains supplementary material, which is available to authorized users.

## Introduction

The effects of rest periods between physical exercises have considerable importance when viewed from the perspective of practice effectiveness as practice efficiency [1]. For instance in continuous tasks fatigue increases and acquisition and retention decreases when the rest period between trials decreases [2]. Therefore the rest periods between trials or exercises may play an important role in rehabilitation to improve performance. Some authors proposed that longer rest periods generally lead to more skill performance during practice [3].

Controversially in the cognitive skill literature, however, between-session delays have been seen either as having a negligible effect on performance or as causing forgetting [4]. In contrast, in the procedural skill literature, overnight between-session delays can result in performance gains [4].

For the motor rehabilitation of patients after stroke there is not much literature about optimal rest periods between exercise sets. In contrast to this, the use of rests should be a very important aspect of daily clinical physiotherapy. Some investigators in gait rehabilitation for example use rather long rest periods between the trials (2–3 min) [5] but other used diametrically short rests of 10 s [6]. Until now no rigorous study has evaluated the immediate effects of different rest periods between common balance exercises after stroke.

The aim of the present study was therefore to investigate the immediate effects of different rest periods on the balance performance of patients after stroke.

## Main text

### Methods

We included all patients after first stroke, aged between 45 and 80 years, who were able to walk with physical assistance or supervision of one person (functional ambulation categories, FAC 2 or 3) [7], reduced muscle strength in the affected hip flexors and abductors (defined as Medical Research Council, MRC grade three or four), without apparent limitations in proprioception of the paretic leg (defined as 3–6 points in the sensory subtest of the Fugl-Meyer Assessment of the lower limb, FMA) [8] and good to moderate trunk control (defined as more than 48 points in the Trunk Control Test, TCT) [9] and written informed consent.

We excluded patients with neurological diseases such as dementia or brain tumours, with orthopaedic disease causing pain in the lumbar spine and hip area, severe global aphasia and a pronounced neglect (defined as ≤ 100 points in the Behavioural Inattention Test, BIT) [10].

We conducted this study with the approval of the local ethics committee of the University of Applied Sciences Gera, Germany (Reference Number 12-2/01/02/01) and with the understanding and written consent of each patient. The study was registered retrospectively in the German Register of Clinical Trials with the ID: DRKS00013979).

Based on our a priori sample size calculation we estimated a required sample size of 20 patients for rejecting the null hypothesis (assumptions were α = 0.05, power = 80% and an assumed difference between groups of 4 s in one-leg standing time (without support). We therefore prepared 20 lots in sealed and opaque envelopes in an urn [10 lots indicating short rest (SR) and ten lots indicating full rest (FR) group] for (concealed) randomisation.

We designed and prepared the patient exercise booklet and used the homepage http://www.physiotherapyexercises.com/ to standardise our seven balance exercises for both the (SR) and (FR) group. We used standardized balance exercises for all patients and the individual progressions and detailed variations for less or more advanced patients are described in Additional file 1: Appendix, in an exercise booklet. According to individual balance difficulties (less and more advanced balance abilities) five out these seven tasks were selected by a physiotherapist as an exercise set and every set lasted for 2 min. The choice of exercises and the increase of the degree of difficulty were adapted to the individual balance abilities of the patient during practice in the sense that they are always challenging for the patient. Therefore all patients had the same type of balance exercises, adopted for their individual balance activities and all exercises were supervised by one physiotherapist. For example, if one patient could not stand on one leg while the other leg was resting on a foam cup, the exercise was adopted and the hands could be used as far as necessary to support the balance of the patient.

Only the duration of rest period between the exercise sets differed between both groups. Physiotherapists with an experience of more than 10 years in stroke rehabilitation instructed patients in the SR group to rest for 1 min after every exercise set and those patients in FR group to rest for 4 min between the sets.

We used the following dependent variables.

- One-leg standing time in seconds (OLST) [11]. We used the OLST on the affected leg with the patients having their eyes open as an index of postural stability, with a maximum time of 60 s allowed.
- Tandem standing time in seconds (TST) [12]. We used the TST with the patients having their eyes open to measure static double stance balance. With this test we measured whether and how long the patient can maintain a tandem standing position (one foot in front of the other foot) without holding on, with a maximum time of 60 s allowed.

We measured the OLST and the TST immediately after the last of their six sets of balance exercises and did retention test of the OLST and the TST 24 h later.

The personnel undertaking the tests and collecting the data and also the statistician did not know to which group the patient was allocated (blinded assessors). However, the therapist who instructed the exercises had to know the group allocation for appropriate commands and rest period.

We present our results as means with standard deviation if not stated otherwise. We presented the results of both groups graphically with individual data using boxes and whiskers. We used SAS/STAT 9.3 for all statistical procedures (SAS Institute Inc., Cary, NC, USA) and statistical assumptions were tested with the implemented functions.

We used always nonparametric tests, e.g. the Mann–Whitney U-test to compare baseline measures and differences between groups. The global alpha level was set at 0.05.

### Results

From July 2014 to March 2015 we screened 43 patients for eligibility in our inpatient rehabilitation centre (see flowchart, Additional file 2). Twenty subjects were eligible and fulfilled our inclusion criteria. Ten patients were randomly allocated in SR group and ten patients in the FR group. All patients received and completed the intervention as planned a priori (see flow chart, Additional file 2). At study onset groups did not differ in important prognostic variables (as shown in Table 1).

**Table 1:** Characteristics of patients at baseline (20 patients; group 1 = 10 patients and group 2 = 10 patients)

	Short rest group	Full rest group	p-value
Age in years, mean (SD)	68.2 (11.91)	62.5 (10.97)	0.10
Gender (male/female)	6/4	8/2	0.62
Type of stroke (ischaemic/haemorrhagic)	9/1	9/1	1.0
Side (left/right)	9/1	7/3	0.58
Duration of illness in weeks, mean (SD)	5.0 (2.58)	5.5 (4.1)	1.00
Berg Balance Scale score in points, mean (SD)	25.3 (16.72)	23.1 (15.14)	0.77
OLST at baseline in seconds, mean (SD)	7.1 (6.68)	2.4 (1.59)	0.09
TST at baseline in seconds, mean (SD)	19.3 (26.31)	11.8 (12.36)	0.60

*SD* standard deviation, *OLST* one-leg standing time in seconds, *TST* tandem standing time in seconds

**Fig. 1:**
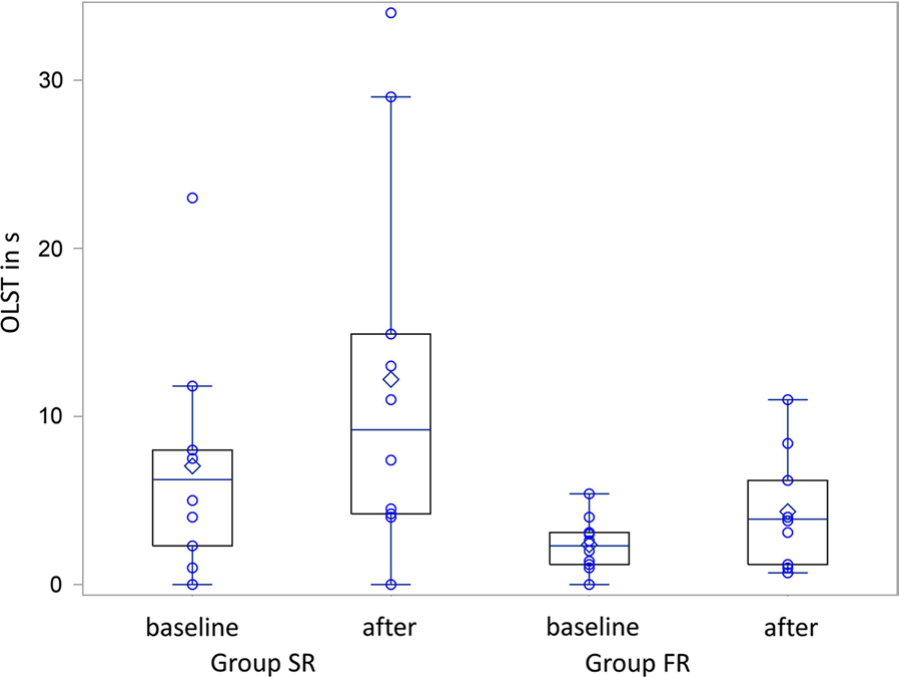
Box and whisker plots showing the results of the OLST in seconds with individual data for each group, before and after the exercises

**Fig. 2:**
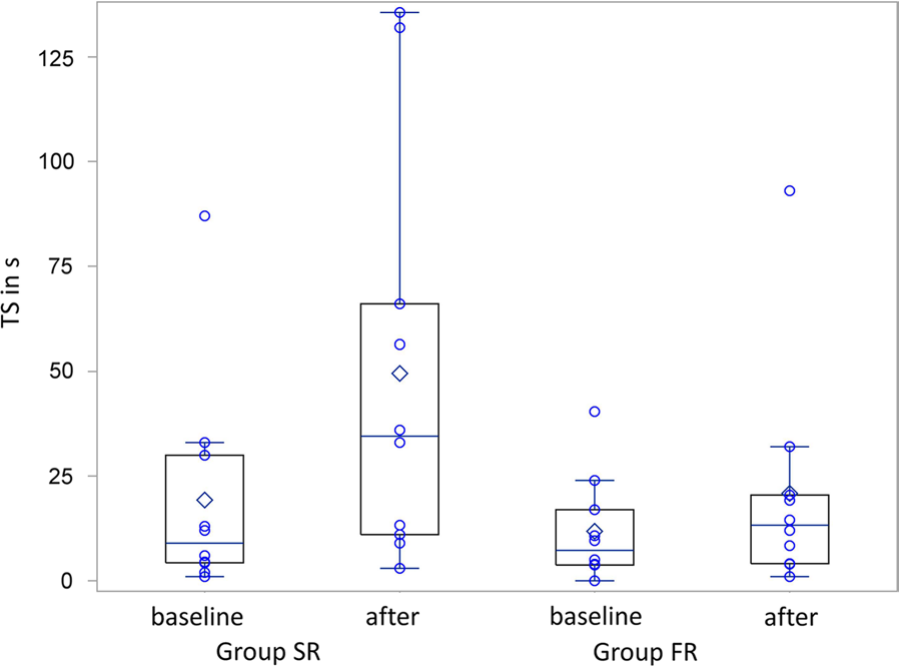
Box and whisker plots showing the results of the TST in seconds with individual data for each group, before and after the exercises

Patients improved from baseline until immediately after balance exercises in OLST [SR: mean difference 5.1 s (SD 10.3) and FR: 2.0 s (2.4), Fig. 1] and TST [SR: 14.9 s (SD 24.6) and FR: 5.7 s (12.0), Fig. 2], but OLST and TST did not differ significantly between groups (p = 0.35 and p = 0.52, respectively).

There was a moderate relationship between the initial values in OLST and TST compared to the results immediately after the exercises (ρ = 0.625 and ρ = 0.716 respectively).

Patients improved from baseline until the retention test 24 h later in OLST [SR: mean difference 10.6 s (SD 20.4) and FR: 1.6 s (3.3)] and TST [SR: 14.5 s (SD 25.5) and FR: 4.2 s (14.9)], but OLST and TST did not differ significantly between groups (p = 0.07 and p = 0.19, respectively).

### Discussion

Our study results did not show any significant differences in one leg and tandem stance performance when comparing different rest periods of 1 min versus 4 min in a single balance exercise session. Neither immediately after exercises nor 24 h later we were able to find any group differences.

This can be interpreted as that using a shorter rest between balance exercises compared to a full rest does not affect the balance performance in OLST and TST after stroke. Since shorter rest periods give the opportunity of applying a higher dose of therapy they therefore should be preferred.

In the peer review literature there is so far hardly any indication of the optimal pause duration within an exercise session for patients after stroke. We set the pause duration so that one group got 1 min pause, the other group 4 min pause between exercises. This is based on other studies that investigated, e.g. the effects of gait therapies after stroke. For example some researcher used rather long rest periods between the trials (2–3 min) [5] but others used short rests of 10 s [6]. Some studies even do not describe exactly how long the pause was. For example a very recent study used backward walking training after stroke to improve balance and described rest breaks by patients’ tolerance in terms of vital signs [13].

Many physiotherapists choose the rest period between sets of exercises based on their own clinical experience in certain situations of therapy. This cannot be seen as automatically wrong or even inappropriate, especially since there is not any hint how long a rest period in the rehabilitation of patients after stroke should definitely last, neither from the peer reviewed literature nor from clinical guidelines.

We found a moderate correlation between the baseline values in OLST and TST compared to the results immediately after the exercises. This means that the patients who had a considerably reduced balance at the beginning also showed lower effects in the follow-up examination.

It is not clear what this individual improvement of the balance in OLST and TST means for the independence of patients after stroke in real everyday life. A recent study found a robust association of balance abilities measured with the Berg Balance Scale’s (BBS) with transfer and stair-climbing independence and performance [14]. The authors calculated cut-off values for independent transfer and stair-climbing for the BBS of 41/40 and 54/53 points, respectively [14]. Our patients had BBS levels well below these limits, demonstrating the severity of our patients’ balance deficit in this study. So far, however, there have been only few randomized studies including severely affected patients. One study included patients with severe balance deficits and used two different attention strategies [15]. They found that the use of an external focus leads immediately to an increased lateral weight shift in a seated position [15], but the authors did not make any statements about the balance in a standing position.

The relatively wide distribution of patients with regard to their balance abilities must also be taken into account in this study. We therefore used a standardized protocol for one exercise session, which seems to be very useful for improving the balance of both mildly and severely affected patients and which can be easily adapted according to the balance abilities of individual patients after stroke.

Other studies used further approaches such as circuit based exercises, strength training or backward walking training or mirror therapy to improve balance after stroke [13, 16–18]. It can therefore be argued that our balance exercises were somewhat static and not dynamic. However, we chose exactly these exercises for our study in order to have an easy to understand and standardized exercise plan for moderate to severely affected patients. The primary interest of our study was not to evaluate the effects of a balance therapy program but to investigate the specific effects of the pause duration.

It can be argued that perhaps one group did more demanding exercises than the other group. Since the balance level at the beginning of the two groups did not differ significantly, we assume that the exercise selection did not differ significantly between the two groups either.

## Limitations

- We included only patients who were able to stand alone without any manual assistance and who were able to do challenging balance exercises while standing.
- Our results are not applicable to patients with very severely affected balance after stroke who cannot stand alone.
- Our results should be seen as a first pilot trial, the sample size was small, only 1 and 4 min pauses were compared and the study encompassed just a single therapy session and hence should be interpreted with caution.
- We collected only data on measures such as OLST and TST before, immediately after and 24 h after the training session.

## Additional files

**Additional file 1.** Balance exercise booklet. No data, a booklet of exercises provided is given.

**Additional file 2.** Flow chart. CONSORT flow-chart of the study.

## Abbreviations

SD: standard deviation
SR: short rest
FR: full rest
OLST: one-leg standing time on the affected leg
TST: tandem standing time
FAC: functional ambulation categories
MRC: Medical Research Council
FMA: Fugl-Meyer Assessment of the lower limb
TCT: Trunk Control Test
BBS: Berg Balance Scale
