# Supplementary material for: Immediate effects of rest periods on balance control in patients after stroke. A randomized controlled pilot trial
Source: BMC Res Notes. 2018 May 24;11:338. doi: 10.1186/s13104-018-3450-2 (PMC5968623; doi:10.1186/s13104-018-3450-2)
Supplement: Supplementary file 1 — Additional file 1. Balance exercise booklet. No data, a booklet of exercises provided is given. [file 13104_2018_3450_MOESM1_ESM.pdf]

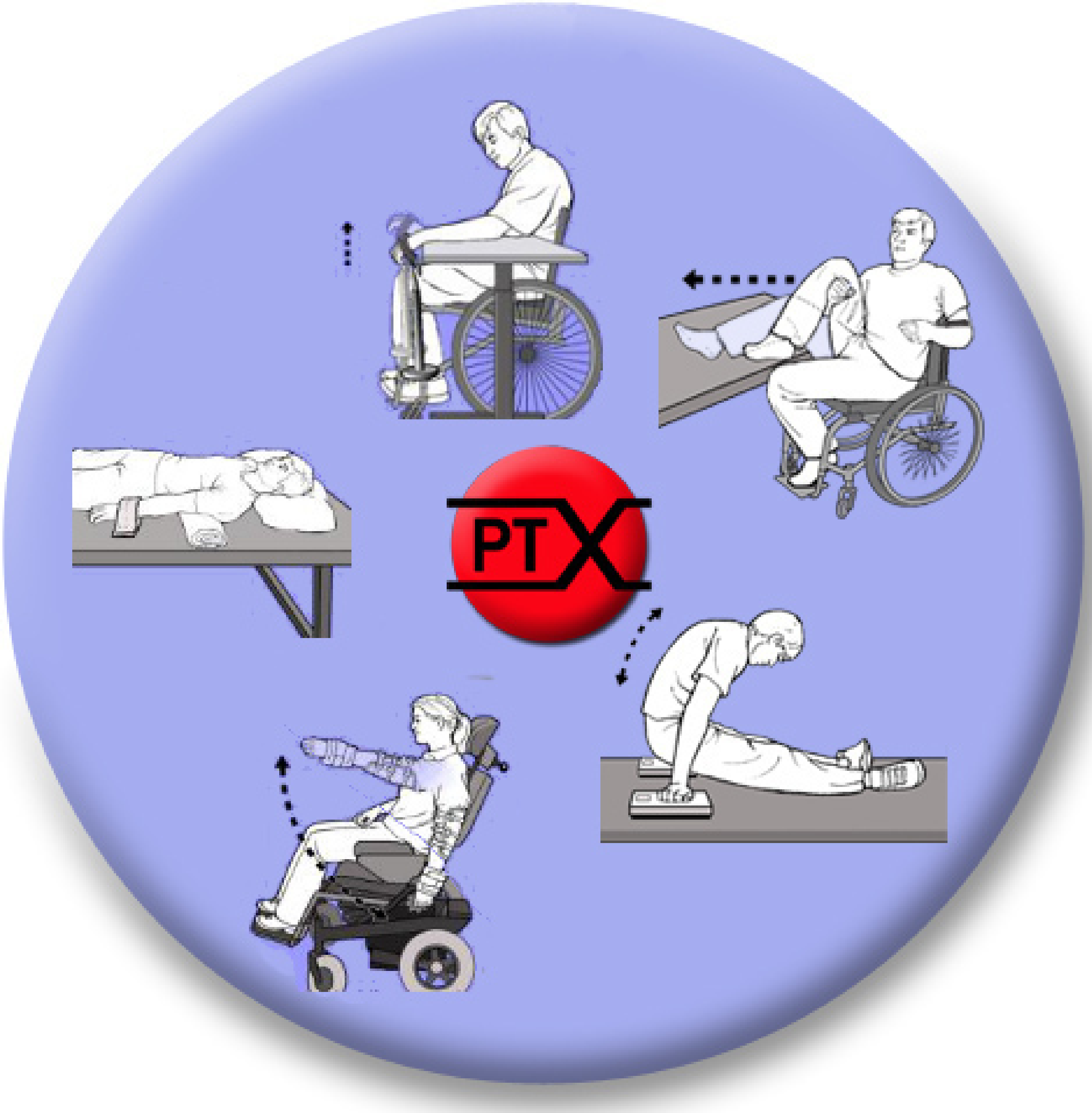

www.physiotherapyexercises.com

Exercise Booklet

Stand and look behind

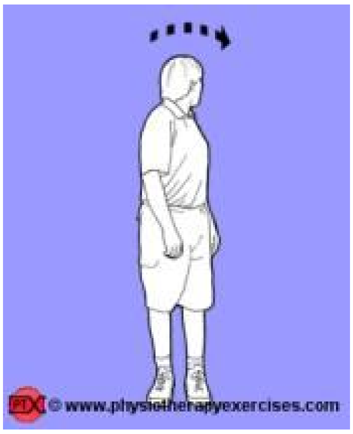

Therapist's aim
To improve the ability to stand and balance.

Client's aim
To improve your ability to stand and balance.

Therapist's instructions
Position the patient in standing with their feet slightly apart. Instruct the patient to turn and look over their shoulder. Ensure that the patient makes a large amplitude movement without moving their feet or taking a step.

Client's instructions
Position yourself standing with your feet slightly apart. Practice turning your head to look over your shoulder. Aim to look around behind you as far as you can, without moving your feet or taking a step.

Progressions and variations
Less advanced: 1. Provide hand support for balance. 2. Decrease the amplitude of the movement. More advanced: 1. Increase the amplitude of the movement. 2. Add concurrent cognitive and/or manual task/s. 3. Stand on one leg.

Stand with one leg forward and look behind

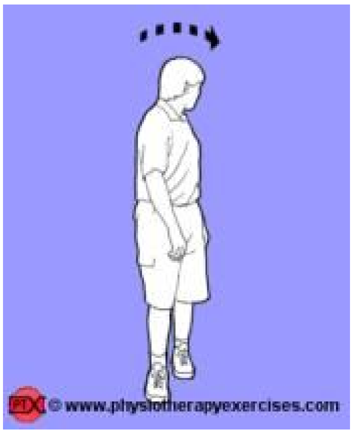

Therapist's aim
To improve the ability to stand and balance.

Client's aim
To improve your ability to stand and balance.

Therapist's instructions
Position the patient in standing with one leg in front of the other. Instruct the patient to turn and look over their shoulder as far as they can. Ensure the patient makes a large amplitude movement without moving their feet or taking a step.

Client's instructions
Position yourself standing with one leg in front of the other. Practice turning to look over your shoulder. Aim to look around behind you as far as you can, without moving your feet or taking a step.

Progressions and variations
Less advanced: 1. Provide hand support for balance. 2. Stand with feet level and slightly apart. 3. Decrease the amplitude of the movement. More advanced: 1. Increase the amplitude of the movement. 2. Add concurrent cognitive and/or manual task/s. 3. Stand on one leg.

Standing on one leg with the other leg resting on a foam cup

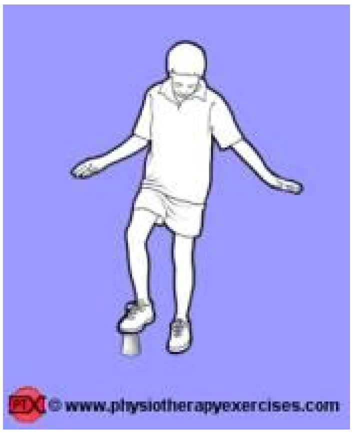

Therapist's aim
To improve the ability to weight-bear through the leg.

Client's aim
To improve your ability to weight-bear through your leg.

Therapist's instructions
Position the patient in standing with a foam cup on the floor in front of them. Instruct the patient to stand on one leg and hold their other foot just resting on the cup. Ensure that the foam cup is not squashed.

Client's instructions
Position yourself standing with a foam cup on the floor in front of you. Practice standing on one leg while holding your other foot just resting on the cup. Ensure that you do not squash the foam cup.

Progressions and variations
Less advanced: 1. Provide hand support for balance. More advanced: 1. Raise and lower the foot on and off the cup. 2. Swap from one leg to the other.

Stand in semi-tandem stance

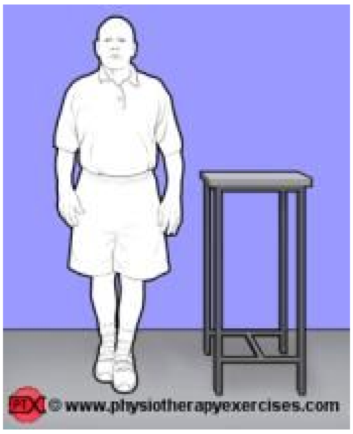

Therapist's aim
To improve the ability to stand and balance.

Client's aim
To improve your ability to stand and balance.

Therapist's instructions
Position the patient in semi-tandem stance with one foot forward, no space between the feet and a stable support nearby. Instruct the patient to maintain the position. Ensure that hand support is used only if necessary.

Client's instructions
Position yourself standing with one foot forward, no space between your feet and a stable support nearby. Practice maintaining the position. Ensure that hand support is used only if you feel unsteady.

Progressions and variations
Less advanced: 1. Position the feet further apart. More advanced: 1. Remove hand support. 2. Turn head and trunk in different directions. 3. Add movement of the arms in different directions. 4. Throw and catch a ball in this position. 5. Stand on one leg. 6. Stand in tandem stance.

Stand in tandem stance

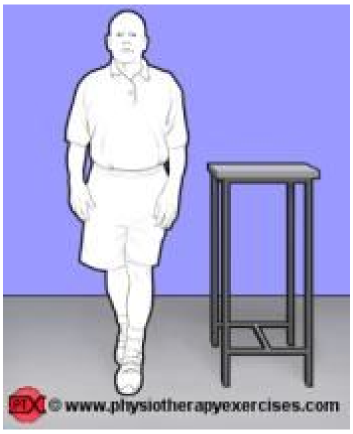

Therapist's aim
To improve the ability to stand and balance.

Client's aim
To improve your ability to stand and balance.

Therapist's instructions
Position the patient in tandem stance with one foot forward in front of the other and a stable support nearby. Instruct the patient to maintain the position. Ensure that hand support is used only if necessary.

Client's instructions
Position yourself standing with one foot forward in front of the other and a stable support nearby. Practice maintaining the position. Ensure that hand support is used only if you feel unsteady.

Progressions and variations
Less advanced: 1. Position the feet in semi-tandem stance. 2. Position the feet further apart. More advanced: 1. Remove hand support. 2. Turn head and trunk in different directions. 3. Add movement of the arms in different directions. 4. Throw and catch a ball in this position. 6. Stand on one leg.

Stand with narrow base of support

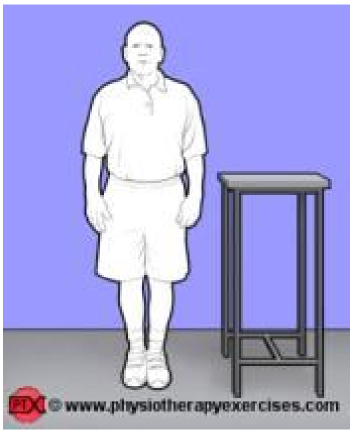

Therapist's aim
To improve the ability to stand and balance.

Client's aim
To improve your ability to stand and balance.

Therapist's instructions
Position the patient in standing with their feet together and a stable support nearby. Instruct the patient to maintain the position. Ensure that hand support is used only if necessary.

Client's instructions
Position yourself standing with your feet together and a stable support nearby. Practice maintaining the position. Ensure that hand support is used only if you feel unsteady.

Progressions and variations
Less advanced: 1. Position the feet further apart. More advanced: 1. Remove hand support. 2. Turn head and trunk in different directions. 3. Add movement of the arms in different directions. 4. Throw and catch a ball in this position. 5. Stand on one leg.

Stand on one leg with support

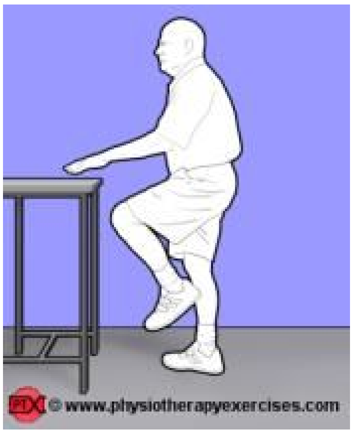

Therapist's aim
To improve the ability to stand on one leg and balance.

Client's aim
To improve your ability to stand on one leg and balance.

Therapist's instructions
Position the patient in standing on one leg with a stable support nearby. Instruct the patient to maintain the position without touching their other foot on the floor. Ensure that hand support is used only if necessary.

Client's instructions
Position yourself standing on one leg with a stable support nearby. Practice maintaining the position without touching your other foot on the floor. Ensure that hand support is used only if you feel unsteady.

Progressions and variations
Less advanced: 1. Stand with both feet on the floor. More advanced: 1. Remove hand support. 2. Turn head and trunk in different directions. 3. Add movement of the arms in different directions. 4. Throw and catch a ball in this position.

Precautions
1. Ensure that the support is stable.

		Mon	Tue	Wed	Thu	Fri	Sat	Sun
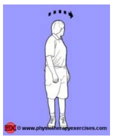	Wk 1							
	Wk 2							
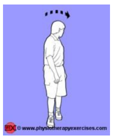	Wk 1							
	Wk 2							
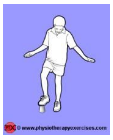	Wk 1							
	Wk 2							
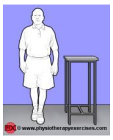	Wk 1							
	Wk 2							
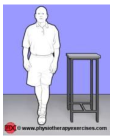	Wk 1							
	Wk 2							
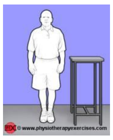	Wk 1							
	Wk 2							
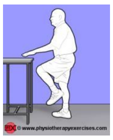	Wk 1							
	Wk 2							
