# Supplementary material for: Immediate effects of rest periods on balance control in patients after stroke. A randomized controlled pilot trial
Source: BMC Res Notes. 2018 May 24;11:338. doi: 10.1186/s13104-018-3450-2 (PMC5968623; doi:10.1186/s13104-018-3450-2)
Supplement: Supplementary file 2 — Additional file 2. Flow chart. CONSORT flow-chart of the study. [file 13104_2018_3450_MOESM2_ESM.doc]

**Flow-chart (according to CONSORT,** [**www.consort-statement.org**](http://www.consort-statement.org/)**)**

Randomised (n = 20)

Allocated to group 1 (n = 10)

short rest (SR)

Received allocated

intervention (n= 10)

Allocated to group 2 (n = 10)

full rest (FR)

Received allocated

intervention (n=10 )

analysed (n = 10)

analysed (n = 10)

Assessed for eligibility (n = 43)

Excluded (n =23)

Early discharge (n = 11)

Second stroke (n = 1)

Not meeting

inclusion criteria (n = 11)
